# Dietary flavonoid myricetin inhibits invasion and migration of radioresistant lung cancer cells (A549‐IR) by suppressing MMP‐2 and MMP‐9 expressions through inhibition of the FAK‐ERK signaling pathway

**DOI:** 10.1002/fsn3.1495

**Published:** 2020-03-09

**Authors:** Hye R. Kang, Jeong Y. Moon, Meran K. Ediriweera, Yeon W. Song, Moonjae Cho, Dharanibalan Kasiviswanathan, Somi K. Cho

**Affiliations:** ^1^ Interdisciplinary Graduate Program in Advanced Convergence Technology and Science Jeju National University Jeju Korea; ^2^ Subtropical/Tropical Organism Gene Bank Jeju National University Jeju Korea; ^3^ Faculty of Biotechnology College of Applied Life Sciences SARI Jeju National University Jeju Korea; ^4^ Department of Biochemistry School of Medicine Jeju National University Jeju Korea; ^5^ School of Biomaterial Science and Technology College of Applied Life Sciences Jeju National University Jeju Korea

**Keywords:** FAK signaling pathway, MMP‐2 and 9, myricetin, transcriptome analysis

## Abstract

Myricetin is a commonly found dietary flavonoid. In the present study, we investigated the effects of myricetin on migration and invasion of radioresistant lung cancer cells (A549‐IR). Transcriptome analysis of A549‐IR cells identified several differentially expressed genes (DEGs) in A549‐IR cells compared to parental A549 cells. Functional enrichment analysis revealed that most of the DEGs were linked with PI3K‐AKT signaling, proteoglycans, focal adhesion, and ECM–receptor interactions. A549‐IR cells demonstrated enhanced migratory potential with increased expression of vimentin, snail and slug, and reduced expression of E‐cadherin. A549‐IR cells exposed to myricetin displayed reduced migration and suppressed MMP‐2 and MMP‐9 expression. Notably, myricetin inhibited the phosphorylation of focal adhesion kinase (FAK) and altered the F‐actin/G‐actin ratio in A549‐IR cells, without modulation of EMT markers. These findings suggest that myricetin can inhibit migration of A549‐IR cells by suppressing MMP‐2 and MMP‐9 expressions through inhibition of the FAK‐ERK signaling pathway.

## Graphical abstract

Transcriptome analysis of radioresistant lung cancer cells (A549‐IR) identified several differentially expressed genes compared to A549 parental cells. Myricetin inhibited invasion and migration of A549‐IR cells by reducing the expression of matrix metalloproteinases (MMP‐2 and MMP‐9) through inhibition of the FAK‐ERK signaling pathway.

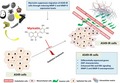

## 1 INTRODUCTION

Lung cancer remains as a major health issue among men and women worldwide. In the United Sates, lung cancer is the second major cause of cancer death in men (Siegel, Miller, & Jemal, 2019). Cigarette smoking is believed to be a major driving force in the development of lung cancer. Of the two main types (non‐small‐cell lung cancer [NSCLC] and small‐cell lung cancer [SCLC]), NSCLC has been reported as the most common histological type (Johnson, 2000). Majority of NSCLC cases are treated with chemo‐ and radio‐therapies. Radiotherapy is considered as a standard treatment modality for unresectable lung cancer (Molina, Yang, Cassivi, Schild, & Adjei, 2008). Nevertheless, several clinical studies have shown that radiotherapy could lead to cancer metastasis and secondary malignancies, causing  a major barrier in lung cancer treatments (Anderson & Dische, 1981; Fagundes, Perez, Grigsby, & Lockett, 1992; Strong et al., 1978). Previous studies have shown that ionizing radiation can stimulate the secretion of numerous cytokines and matrix metalloproteinases (MMPs) acting through the PI3K/AKT or MAPK pathways (Cheng, Chou, Kuo, & Hsieh, 2006; Cui et al., 2015). Furthermore, transforming growth factor (TGF)‐β has been reported to play a key role in determining the cancer cell motility upon ionizing radiation (Carl et al., 2016). Although ionizing radiation has been reported to stimulate EMT and cancer cell invasion, molecular mechanisms associated with radiation‐induced cancer invasion remain debatable.

Epithelial–mesenchymal transition (EMT) and its opposite process, mesenchymal–epithelial transition (MET), are vital embryonic processes. EMT, which is well known as an activated developmental program during cancer invasion and metastasis, often occurs at the invasive front of many metastatic cancers (Wang & Zhou, 2011). EMT leads to the loss of cell‐to‐cell interactions, repression of E‐cadherin expression and induction of vimentin expression, thereby resulting increased cancer cell mobility (Peinado, Olmeda, & Cano, 2007). As epithelial cancer cells undergo EMT, they acquire metastatic ability. EMT is reported to be controlled by several transcription factors including snail, slug twist, and Zeb1 (Nieto, 2002). The zinc‐finger transcriptional factors snail and slug have been characterized as the key EMT regulators and E‐cadherin repressors (Zhao & Guan, 2009). Focal adhesion kinase (FAK) is mainly involved in cellular signal transduction in a range of cells (Schaller, 2010). Moreover, FAK has also been found to involve in cell adhesion and migration (Zhang et al., 2014). Overexpression of FAK has been detected in various cancer types (Avizienyte & Frame, 2005; Golubovskaya et al., 2014; Mael‐Ainin, Abed, Conway, Dussaule, & Chatziantoniou, 2014). Furthermore, accumulating evidence demonstrates that FAK can play an important role in EMT (Cicchini et al., 2008). A study by Bouchard et al., 2007, demonstrated that FAK can regulate PI3K/AKT signaling, causing upregulation of vimentin and snail.

Myricetin is a natural flavonoid which is abundantly found in vegetables, fruits, teas, and some medicinal plants. Several studies have illustrated that myricetin can exert anti‐oxidant, anti‐inflammatory, and anti‐cancer effects (Boam, 2015; Gordon & Roedig‐Penman, 1998; Lu et al., 2006; Roedig‐Penman & Gordon, 1998; Sun et al., 2012; Wang et al., 2010). Moreover, myricetin has been reported to show chemosensitizing effects (Huang et al., 2015; Yi et al., 2015). Given the influence of radiotherapy in the enhancement of metastatic ability in cancer patients, establishment of new in vitro models to investigate preclinical efficacy of novel drug leads on reversing radiation‐induced metastasis will underpin cancer therapeutics. Thus, in this investigation, radiation‐induced metastatic cancer cell line (A549‐IR) was established from parental A549 lung cancer cells, and metastasis inhibitory effects of myricetin in A549‐IR cells were examined to provide alternative cancer therapeutic approaches to improve the life expectancy of NSCLC patients.

## 2 MATERIALS AND METHODS

### 2.1 Reagents

Ham's F‐12 medium, antibiotics solution, Alexa Fluor 488‐phalloidin, and trypsin‐EDTA were purchased from the Invitrogen Inc. Fetal bovine serum (FBS) was purchased from the Corning Incorporated‐Life Sciences. MTT and DMSO were purchased from the Amresco Inc. Gelatin and Bradford reagent were purchased from the Sigma Chemical Co. Primary antibodies were used at 1:1,000 dilution, except β‐actin (1:10,000). HRP‐conjugated goat anti‐rabbit IgG secondary antibody (Vector Laboratories) was used at 1:5,000 dilution. BS ECL Plus Kit (Biosesang, Inc.) was used to detect protein bands.

### 2.2 Cell culture and establishment of irradiation‐tolerant cell lines

To establish radiation‐tolerant cell lines, a previously reported methods were used with slight modifications (Carl et al., 2016; Cui et al., 2015; Ishihara et al., 2010; Jing et al., 2009). A549 cells (6 × 10^4^ cells/ml in 1 ml PBS) were subjected to irradiation with a single fraction to generate irradiation‐tolerant cell lines. The irradiation was performed using a ^60^CO Theratron‐780 teletherapy unit (Applied Radiological Science Institute, Jeju National University, Korea) at a dose rate of 1.43 Gy per min. Cells were placed at room temperature for <30 min during irradiation. After irradiation, cells were immediately seeded in a culture flask (60‐mm‐culture flask) and incubated at 37°C in a humidified incubator with 5% CO_2_. Irradiated cells were allowed to grow for 15 days (fresh medium was provided every 3–4 days). Following incubation, surviving clones from 12 Gy‐irradiated cells were transferred to each well of a 96‐well culture plate. Upon subconfluency, each single clone was transferred to large scale culture flasks, 48‐well, 24‐well, 12‐well, and 6‐well plates. Finally, surviving clones from each irradiated cells were established and designated as A549‐IR cell line.

### 2.3 Cell viability

Effects of the testing agents on A549 or A549‐IR cell viability were assessed using the MTT colorimetric assay. For the MTT assay, cells (4 × 10^3^ cells/well) were cultured in 96‐well culture plates. After 24‐hr incubation, cells were treated with various concentrations of testing agents and incubated for 48 hr. Following incubation, 20 μl (5 mg/ml) of MTT solution was added to each well of the plates and incubated at 37°C for 4 hr. Following incubation, medium was removed and DMSO was added to each well of the plates. Absorbance was recorded at 570 nm using a microplate reader. After calculating cell viability percentages, IC_50_ values were determined using the GraphPad Prism software.

### 2.4 Wound healing assay

Cells (2 × 10^5^ cells/well) were cultured in 12‐well cell culture plates. Upon forming confluent monolayers, wounds were created using a sterile tip. Cells were then washed with PBS and treated with testing agents. Wounds were photographed using a phase microscopy at 0‐, 24‐, and 48‐hr incubations to assess the cell migration, and percentage of wound closure was then determined.

### 2.5 Gelatin zymography analysis

Cells (3 × 10^5^ cells) were seeded in 100‐mm plates and cultured overnight. After incubation, cells were exposed to myricetin in FBS‐free media for 48 hr. Conditioned media were then collected by centrifuging at 7,500*g* for 25 min at 4°C. Concentrated media were stored at −70℃ until used. Protein quantity was determined using the Bradford reagent. MMP‐2 and MMP‐9 enzymatic activities were evaluated by gelatin zymography according to a method described previously (Troeberg & Nagase, 2003). Briefly, electrophoresis was performed under nonreducing conditions on 1% gelatin containing polyacrylamide gels. Following electrophoresis, gels were washed twice with the washing buffer (2.5% Triton X‐100 and 0.02% NaN_3_). Then, gels were again washed with the incubation buffer (50 mM Tris‐HCl, pH 8.0, 5 mM CaCal_2_, and 0.0% NaN_3_) for 10 min. Gels were placed in a sealable container with fresh incubation buffer and incubated at 37°C for 48 hr. After 48‐hr incubation, gels were stained with Coomassie blue solution and MMPs activity was determined.

### 2.6 Western blot analysis

Western blotting was conducted as previously described by us (Moon, Hung, Unno, & Cho, 2018). All the primary antibodies were diluted at 1:1,000 for experiments. HRP‐conjugated anti‐rabbit or anti‐mouse IgG was used at a 1:5,000 dilution. To detect the protein bands, membranes were exposed to X‐ray films and bands were identified using the BS ECL Plus Kit (Biosesang). The band intensities were measured using ImageJ software.

### 2.7 Quantitative real‐time PCR analysis

TRIzol™ reagent (Invitrogen) was used to extract RNA from cells. Reverse Transcription Kit (Promega) was used to synthesize complementary DNA (C‐DNA) from 1 µg of RNA. Quantitative real‐time PCR was carried out using the TOPreal™ qPCR preMIX (Enzynomics).

### 2.8 Transcriptome analysis

Transcriptomic analysis in A549 and A549‐IR cells was performed as described in our recent study (Moon et al., 2018).

### 2.9 Actin polymerization assay

Cells were exposed to myricetin for 24 hr and lysed using an actin extraction buffer. Resulted cell lysates were centrifuged at 21,000*g* for 10 min. Pellets were then subjected to SDS‐PAGE, and Western blotting was carried out as previously described (Moon et al., 2018).

### 2.10 Immunofluorescence

Cells (5 × 10^4^ cells/well) were cultured on cell culture‐treated coverslips until 80% confluency. Cells were then fixed with 4% paraformaldehyde and blocked with PBS (1% bovine serum albumin [BSA]). To stain the filamentous actin (F‐actin) and nucleus, cells were incubated with Alexa Fluor 488‐phalloidin and Hoechst33342, respectively. After staining, cells were washed with PBS and photographed using a laser scanning confocal microscope (Olympus FV1200; Olympus Co‐operation).

### 2.11 Statistical analysis

Three independent experiments were conducted in triplicate, and data are illustrated as means ± *SD*. Data were subjected to one‐way ANOVA analysis using the SPSS ver. 12.0 for Windows. *p* < .05 were considered statistically significant.

## 3 RESULTS

### 3.1 Irradiation‐tolerant A549‐IR cell line displays enhanced invasive/migratory potential

**Figure 1:**
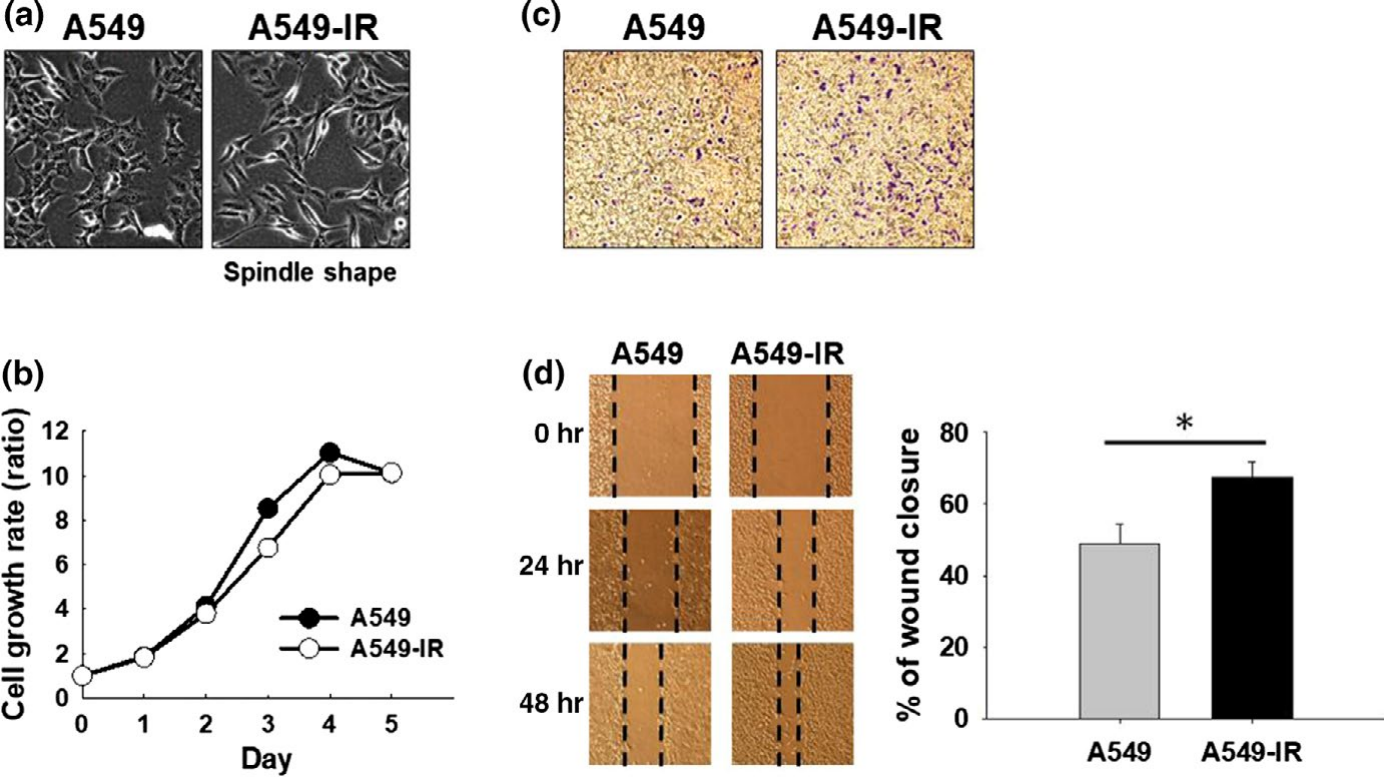
Irradiation promotes invasive and migratory properties of A549 cells. (a) A549 and A549‐IR cells morphology. (b) Cell growth of A549 and A549‐IR cells as measured by the MTT assay. (c) Migration and (d) invasion were analyzed using the transwell invasion/migration assay with same number of A549 and A549‐IR cells for 48 hr. *p < .05

To investigate whether the established irradiation‐tolerant A549 cell line possesses increased migratory potential, cell characteristics were first compared between A549 and 12 Gy‐irradiated A549 cells (A549‐IR). A549‐IR cells displayed spindle‐like appearance (Figure 1a). We next examined the effects of IR on the growth of A549 parental cells. As shown in Figure 1b, the growth rate of A549 cells was not altered significantly upon IR treatment. By performing the invasion assay, we also showed that A549‐IR cells possess more invasive properties compared to A549 cells (Figure 1c). Moreover, A549‐IR cells showed enhanced migratory potential compared to A549 cells (Figure 1d). Taken together, these findings show that irradiation promotes invasive and migratory potential of A549 cells accompanied by cell morphology changes.

### 3.2 Transcriptome analysis

**Figure 2:**
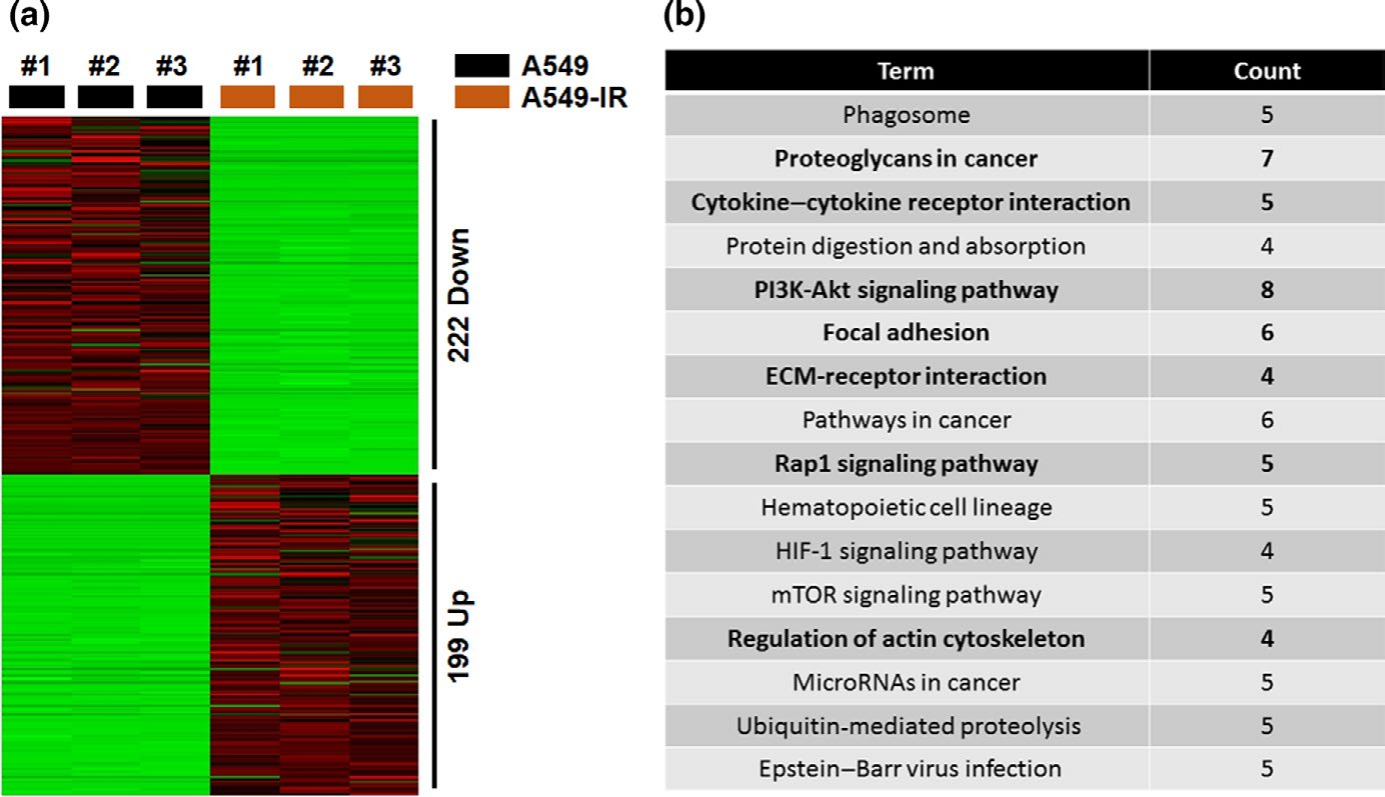
Analysis of differentially expressed genes (DEGs) in A549‐IR cells compared to parental A549 cells. (a) Heatmap plot of DEGs. (b) KEGG categories of DEGs in each pathway versus total number of annotated genes

In order to understand the molecular mechanisms associated with migratory properties of A549‐IR cells, transcriptome analysis was performed. A549‐IR cells displayed an altered global transcription profile, including 199 upregulated and 222 downregulated genes (Figure 2a). Functional enrichment analysis of the DEGs based on the Kyoto Encyclopedia of Genes (KEGG) revealed that most of the regulated genes were associated with proteoglycans in cancer, cytokine–cytokine receptor interaction, PI3K/AKT, focal adhesion, ECM and receptor interactions, Rap1 signaling pathway, actin cytoskeleton regulation, and ubiquitin‐mediated proteolysis (Figure 2b). These results hinted that involvement of EMT‐related activities might be critical in A549‐IR cells.

### 3.3 A549‐IR cells exhibit EMT and express high levels of MMPs

**Figure 3:**
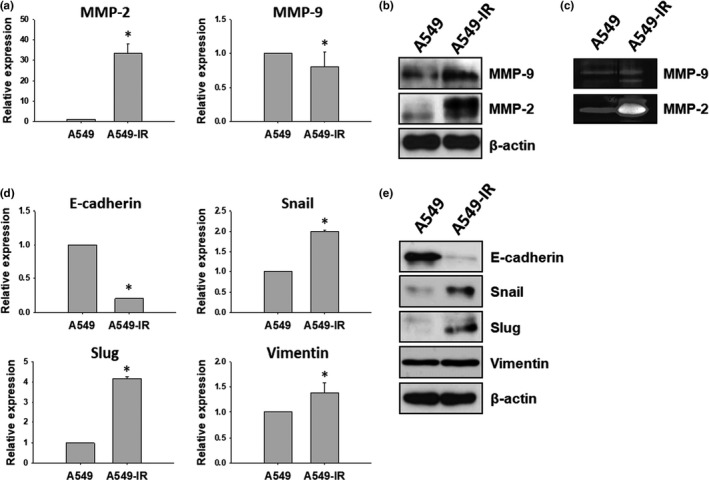
EMT characteristics of A549‐IR cells. (a, b) Expression levels of MMP‐2 and MMP‐9 as measured by qRT‐PCR and Western blot. (c) MMP‐2 and MMP‐9 activities in A549 and A549‐IR cells measured by gelatin zymography analysis. (d, e) EMT‐related genes and proteins (E‐cadherin, snail, slug, and vimentin) expression in A549 and A549‐IR cells examined by qRT‐PCR and Western blot (*p < .05)

MMPs expression in A549 and A549‐IR cells was first measured to check the accuracy of transcriptome analysis. As expected, A549‐IR cells showed increased expression of MMP‐2 gene and protein levels (Figure 3a,b). Interestingly, low expression of *MMP‐9* gene was observed in A549‐IR cells compared to parental cells (Figure 3a). However, overexpressed MMP‐2 and MMP‐9 protein levels were observed in A549‐IR cells (Figure 3b). Results of the gelatin zymography analysis also showed increased levels of MMP‐2 and MMP‐9 in A549‐IR cells (Figure 3c). Of note, expression of EMT‐related genes was also altered in A549‐IR cells. Upregulation of snail, slug and vimentin genes, and proteins and downregulation of E‐cadherin gene and protein were observed in A549‐IR cells (Figure 3d,e). Thus, these results suggest that IR can promote invasion and migration ability of A549 cells by promoting EMT characteristics.

### 3.4 Myricetin inhibits migration of A540‐IR cells

**Figure 4:**
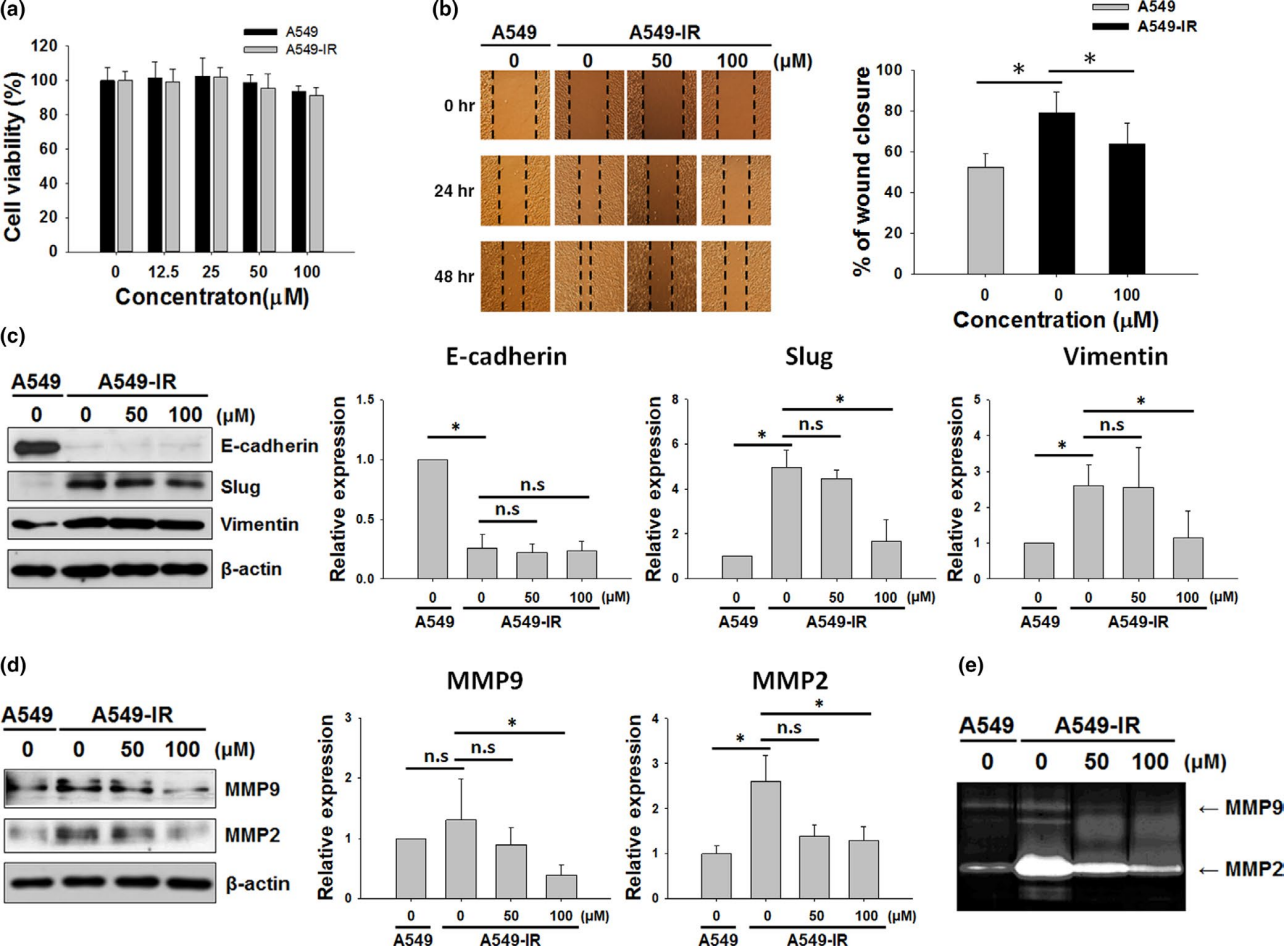
Effects of myricetin on MET in A549‐IR cells. (a) Cytotoxic effects of myricetin in A549 and A549‐IR cells as measured by the MTT assay. Cells were exposed with indicated doses of myricetin for 48 hr prior to the assay. (b) Wound healing assay was performed to assess the effects of myricetin on A549 and A549‐IR cell migration. (c) EMT‐relate proteins (E‐cadherin, slug, and vimentin) expression in A549 and A549‐IR cells after myricetin treatment for 48 hr. (d) MMP‐9 and MMP‐2 expressions in A549 and A549‐IR cells after myricetin treatment for 48 hr. (e) Gelatin zymography analysis was performed to assess MMP‐2 and MMP‐9 levels in A549 and A549‐IR cells following myricetin treatment for 48 hr (n.s: not significant, *p < .05)

Cytotoxic effects myricetin in A549 and A549‐IR cells were evaluated by the MTT assay. After 48 hr post‐incubation, marginally cytotoxic effects were observed in myricetin‐treated A549‐IR cells (Figure 4a). To determine the effects of myricetin on A549‐IR cell migration, we performed the wound healing assay. The result demonstrated that myricetin can significantly inhibit A549‐IR cell migration dose‐dependently (Figure 4b). Therefore, we speculated that the enhanced migration/invasion ability of A549‐IR cells may be inhibited by myricetin treatment. To compare the expression of EMT‐related proteins in A549 and A549‐IR cells and to assess the effects of myricetin on MET in A549‐IR cells, expression of EMT‐related proteins was evaluated after 48‐hr myricetin treatment. Expression of slug, vimentin, MMP‐9, and MMP‐2 was found to increase in A549‐IR cells, while the expression of E‐cadherin was found to decrease (Figure 4c,d). Notably, elevation of E‐cadherin expression was not significant upon myricetin treatment in A549‐IR cells (Figure 4c). In contrast to E‐cadherin, expression of slug, vimentin, MMP‐2, and MMP‐9 was significantly (*p* < .05) reduced at 100 µM myricetin treatment in A549‐IR cells by 2.99‐, 2.28‐, 2.04‐, and 3.30‐fold, respectively (Figure 4c,d). Results of the gelatin zymography analysis also confirmed that myricetin can reduce the expression of MMP‐2 (Figure 4e). These results indicate that myricetin can barely induce MET in A549‐IR cells, while suppressing migration of A549‐IR cells through reducing MMP‐2 and MMP‐9 expression levels.

### 3.5 Myricetin inhibits FAK‐Erk signaling pathway in A549‐IR cells

**Figure 5:**
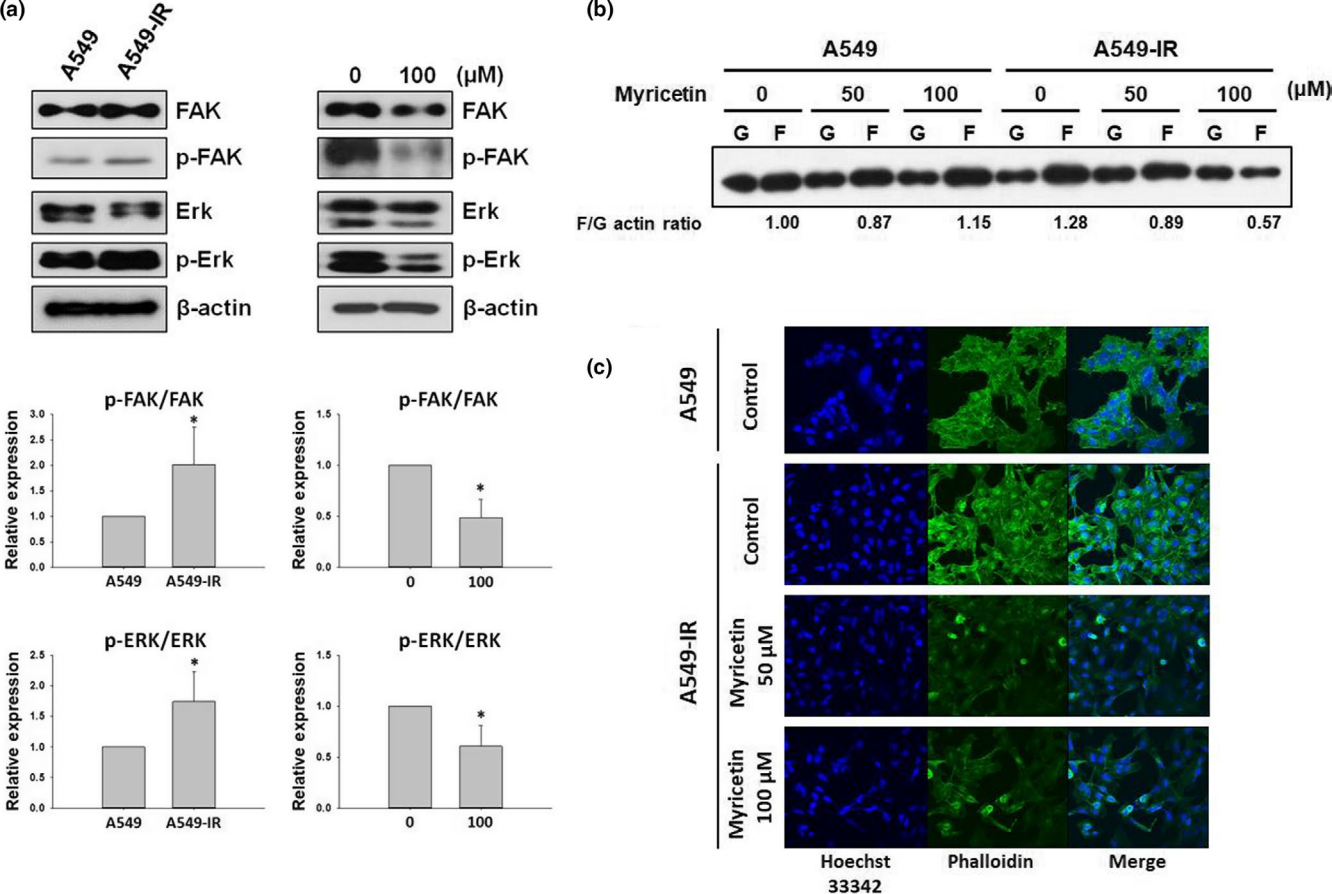
Myricetin regulates actin cytoskeleton remodeling through inhibition of the FAK‐ERK signaling pathway in A549‐IR cells. (a) Effects of irradiation and myricetin on the FAK‐ERK signaling in A549 and A549‐IR cells. (b) Quantification of F/G‐actin ratio in myricetin‐treated A549 and A549‐IR cells. (c) F‐actin stained with Alexafluor 488 phalloidin in A549 and myricetin‐treated A549‐IR cells. *p < .05

According to transcriptome analysis, A549‐IR cells displayed modifications in genes responsible for the actin cytoskeleton‐related pathway (s) (Figure 2). The FAK‐ERK signaling pathway has been reported to involve in cytoskeleton remodeling (Su et al., 2013). Therefore, we first examined the effects of radiation on FAK‐ERK signaling in A549 and A549‐IR cells. Compared to A549 cells, A549‐IR cells showed elevated levels of p‐FAK and p‐ERK (Figure 5a). Following treatment with myricetin, decreased phosphorylation of FAK and ERK was observed (2.07‐ and 1.65‐fold, respectively), suggesting that myricetin can suppress irradiation mediated FAK‐ERK signaling in A549‐IR cells. Moreover, actin polymerization assay was performed to confirm whether inhibition of the FAK‐ERK signaling by myricetin has an effect on actin dynamics. As seen in Figure 5b, F‐actin/G‐actin ratio was decreased upon myricetin exposure in A549‐IR cells. Alexa 488 Fluor™‐conjugated phalloidin staining of F‐actin also illustrated that A549‐IR cells possessed EMT‐like morphology compared to parental A549 cells (Figure 5c).

## 4 DISCUSSION

Lung cancer is a serious health issue in the world (Lowenstein et al., 2019). Over the past two decades, significant efforts have been invested to develop new screening techniques and treatment strategies for lung cancer. Despite the significant efforts invested to improve the  survival of lung cancer patients, lung cancer still ranks as one of the leading cancers worldwide. Chemotherapy along with radiotherapy is recommended as the first‐line treatment for patients with advanced stage or metastasis lung cancer (Lowenstein et al., 2019). Intriguing evidence demonstrates that radiation therapy is not always clinically meaningful as radiation therapy can increase the invasiveness of lung cancer. Moreover, serious side effects have been associated with radiotherapy treatments (Multhoff & Radons, 2012).

More importantly, radiotherapy treatments have been reported to trigger metastasis by inducing EMT, making more adverse consequences in cancer patients (Kawamoto et al., 2012; Liu et al., 2014; Yan et al., 2013). Underlying molecular mechanisms involved in radiation‐induced metastasis and EMT in lung cancer are not fully understood. In the present investigation, we first established a radiation‐resistant lung cancer cell line (A549‐IR) from A545 parental lung cancer cells. EMT marker characterization demonstrated decrease in E‐cadherin expression in A549‐IR cells compared to A549 cells. A549‐IR cells also showed higher expression of slug and vimentin compared to A549 cells. Moreover, MMP‐2 and MMP‐9 expressions, markers for tumor invasiveness, were also increased in A549‐IR cells.

Transcriptome analysis has been reported as an important approach to identify genetic signatures and biological processes associated with human diseases including cancer (Rhodes & Chinnaiyan, 2005). With the availability of advanced next‐generation sequencing (NGS) techniques and modern bioinformatics tools, specific transcriptome profiles have been constructed to understand differentially expressed genes in a range cancer types, making a powerful tool to acquire information related to the genetic basis of cancer (Rhodes & Chinnaiyan, 2005). According to the results of transcriptome analysis, A549‐IR cells showed altered global transcription profile, including 199 upregulated and 222 downregulated genes. Functional enrichment analysis of the DEGs based on the Kyoto Encyclopedia of Genes (KEGG) discovered that most of the genes altered in A459‐IR cells were associated with proteoglycans in cancer, cytokine–cytokine receptor interaction, PI3K/AKT signaling pathway, focal adhesion, ECM receptors, Rap1 signaling pathway, regulation of actin cytoskeleton, and ubiquitin‐mediated proteolysis. We also observed that irradiation triggered actin cytoskeleton remodeling through the FAK‐ERK signaling pathway and promote EMT in NSCLC.

In this study, effects of myricetin, a commonly found secondary metabolite in vegetables, teas, berries and fruits, and some medicinal plants, on invasion and migration of radioresistant lung cancer cells (A549‐IR) were evaluated in vitro. Myricetin exhibited moderate cytotoxic effects in A549‐IR cells in a dose‐dependent manner. Myricetin also inhibited migration of A549‐IR cells dose‐dependently. Among the EMT‐related proteins, expression of MMP‐2, MMP‐9, and slug was downregulated upon myricetin treatment, while the vimentin and E‐cadherin expressions were not altered. E‐cadherin expression has been reported to associate with tumor differentiation and lymph node metastasis in previous clinical studies (Gabbert et al., 1996; Grigoras et al., 2017). Reduced E‐cadherin expression in NSCLC patients has been reported to be link with tumor differentiation, lymph node metastasis, and poor prognosis (Kase et al., 2000; Shibanuma et al., 1998). However, Myong (2004), reported no significant relationships exists between the reduced E‐cadherin expression and survival rate of the NSCLC patients. Similar negative results have also been found in another study showing that the lack of expression of cadherin/catenin complex is not linked with histopathological features of epithelial cancer (Han et al., 1997). Nawrocki et al. (1998) reported a negative correlation between the expression of adhesion molecules and cancer invasion in bronchopulmonary carcinomas, indicating that loss of E‐cadherin is not always associated with early invasion. According to the results of the present study, myricetin did not restore the expression of E‐cadherin reduced by radiation treatments. Instead, it inhibited EMT by suppressing slug, MMP‐2, and MMP‐9 expression, and FAK‐ERK phosphorylation.

In conclusion, results of the present study provide experimental evidence that myricetin can inhibit invasion and migration of radioresistant lung cancer cells (A549‐IR) by suppressing the expression of MMP‐2 and MMP‐9 through inhibition of the FAK‐ERK signaling pathway.

## CONFLICT OF INTEREST

The authors declare no conflict of interest.

## ETHICAL APPROVAL

The authors declare that this study did not involve human or animal subjects, and human and animal testing are unnecessary in our study.
